# Human tolerogenic DC-10: perspectives for clinical applications

**DOI:** 10.1186/2047-1440-1-14

**Published:** 2012-09-28

**Authors:** Giada Amodio, Silvia Gregori

**Affiliations:** 1 San Raffaele Telethon Institute for Gene Therapy (OSR-TIGET), Division of Regenerative Medicine, Stem Cells and Gene Therapy, San Raffaele Scientific Institute, Via Olgettina, 58, 20132, Milan, Italy

**Keywords:** IL-10, Dendritic cells, Tolerance, Type 1 regulatory T cells, Regulatory Antigen-Presenting Cells

## Abstract

Dendritic cells (DCs) are critically involved in inducing either immunity or tolerance. During the last decades efforts have been devoted to the development of *ad hoc* methods to manipulate DCs *in vitro* to enhance or stabilize their tolerogenic properties. Addition of IL-10 during monocyte-derived DC differentiation allows the induction of DC-10, a subset of human tolerogenic DCs characterized by high IL-10/IL-12 ratio and co-expression of high levels of the tolerogenic molecules HLA-G and immunoglobulin-like transcript 4. DC-10 are potent inducers of adaptive type 1 regulatory T cells, well known to promote and maintain peripheral tolerance. In this review we provide an in-depth comparison of the phenotype and mechanisms of suppression mediated by DC-10 and other known regulatory antigen-presenting cells currently under clinical development. We discuss the clinical therapeutic application of DC-10 as inducers of type 1 regulatory T cells for tailoring regulatory T-cell-based cell therapy, and the use of DC-10 as adoptive cell therapy for promoting and restoring tolerance in T-cell-mediated diseases.

## Introduction

Dendritic cells (DCs) are potent antigen-presenting cells (APCs) that possess the ability to stimulate naïve T cells [1]. They represent an essential link between innate and adaptive immunity and are widely distributed in all tissues, especially in those that provide an environmental interface. DCs develop from bone marrow progenitors and circulate in the bloodstream as immature precursors prior to migration into peripheral tissues. DCs patrol the extracellular milieu, and uptake and process antigens (Ags), subsequently presenting them on the cell surface in complex with major histocompatibility molecules. Upon appropriate stimulation, DCs undergo maturation and migrate to secondary lymphoid organs where they present Ags to T cells and prime adaptive immunity. In the steady state, immature DCs migrate at low ratio to the lymph nodes, without undergoing activation, where they can thereby present Ags to T cells in the absence of co-stimulation and induce clonal T-cell anergy [2] or regulatory T cells (Tregs) [3].

It is generally accepted that DCs implicated in tolerance are in a different state of activation and/or differentiation. The microenvironment in which DCs reside and are activated may affect their functions towards tolerance rather than active immune response. However, it has become evident that specialized subsets of DCs, identified according to the expression of specific markers, promote and maintain tissue homeostasis and tolerance. One example are Langherans cells characterized by the expression of langerin (CD207) and birbeck granules [4,5], which represent a specialized subset of immature DCs resident in the skin. It has been shown that inflammatory stimuli can either promote the diffe-rentiation of Langherans cells that initiate a productive immune response or their recruitment as immature DCs into the T-cell areas of lymph nodes where they contri-bute to tolerance [4]. Another example of tolerogenic DCs are that express CD103 reside in the lamina propria of the small intestine [6,7]. Similarly to murine CD103^+^ DCs, [6,8,9], human CD103^+^ DCs isolated from mesenteric lymph nodes have been shown to promote Tregs and to control tissue homeostasis [4]. In addition, a subset of plasmacytoid DCs co-expressing CD123 and CCR6 can be identified in draining lymph nodes of melanoma-bearing patients [10]. These DCs are characterized by the expression of indoleamine-2,3-dioxigenase (IDO) and have been shown to control immune responses *in vitro*.

During the last decades, several molecules that modulate DC functions toward tolerance have been identified [11], providing the possibility to exploit their use *in vitro* for the generation of tolerogenic DCs. Different cytokines have been used during differentiation or activation of DCs, such as TNFα [12,13], granulocyte–macrophage colony-stimulating factor (GM-CSF) [14], granulocyte colony-stimulating factor (G-CSF) [15,16], macrophage colony-stimulating factor (M-CSF) [17], hepatocyte growth factor (HGF) [18], IL-10 alone [19-21] or in combination with transforming growth factor beta (TGFβ) [22]. Alternatively, pharmacological mediators, including 1,25-dihydroxyvitamin D3 [23,24], glucocorticoids [25], prostaglandin E_2_[26-28], or immunosuppressive drugs such as cyclosporine [29], tacrolimus [30], mycophenolate mofetil [31] or rapamycin (RAPA) [32,33] modulate DCs. Tolerogenic DCs can be also generated by culturing monocyte-derived DCs with ligands for immunoglobulin-like transcripts (ILTs; that is, the nonclassical HLA-G molecule [34]), or cobalt protoporphyrin, an inducer of heme oxygenase-1 (HO-1) [35]. Finally, advances in gene-transfer technology offer the possibility to genetically manipulate DCs to endow their tolerogenic potential by overexpressing immunosuppressive molecules such as cytotoxic T-lymphocyte antigen 4, IDO, or IL-10 [11].

The aforementioned strategies target DC differentiation and/or activation and inhibit IL-12 production, thereby limiting the capacity of DCs to prime and/or activate effector T cells. Some of these treatments are also able to promote the upregulation of tolerogenic molecules such as ILTs, IDO, and HO-1, or the secretion of immunomodulatory cytokines, all of which are important for the induction and/or activation of Tregs.

Tregs are specialized subsets of T cells involved in promoting and maintaining immune tolerance *via* their ability to control responses to self and foreign Ags. Over the years, several types of Tregs have been identified but, to date, the best characterized are the forkhead box P3 (FOXP3)-expressing regulatory T cells (FOXP3^+^ Tregs) [36] and the CD4^+^ IL-10-producing type 1 regulatory T (Tr1) cells [37]. FOXP3^+^ Tregs can be either naturally occurring which are selected in the thymus, or adaptive [38]. Tr1 cells can be induced in the periphery upon chronic Ag stimulation in the presence of IL-10 [39], and are currently identified by their unique cytokine profile consisting of high levels of IL-10, TGFβ, low levels of IL-2 and variable amounts of IFNγ, in the absence of IL-4 [37,40]. Depending on the agent used for tolerogenic DC induction, the resulting DCs are equipped with defined tolerogenic molecules, which determine their ability to promote either FOXP3^+^ Tregs or Tr1 cells [41].

In this review we will discuss the role of IL-10 in the induction of human tolerogenic DCs focusing our attention on a subset of tolerogenic DCs, termed DC-10, identified and characterized by our group [21]. The major characteristics of these cells will be compared with those of other tolerogenic APCs currently under clinical development. Foreseen clinical applications of DC-10 will be also discussed.

### IL-10 and modulation of dendritic cells

IL-10 is an immune-modulatory cytokine that plays a central role in controlling inflammation, inhibiting immune responses, and inducing tolerance [42]*.* IL-10 downregulates the expression of major histocompatibi-lity complex class II and co-stimulatory molecules, CD80 and CD86, on DCs [43-45]. In addition, the release of IL-1β, IL-6, TNFα and, most markedly, IL-12 by DCs is abolished after IL-10 treatment [42,46,47]. These effects have been shown either when immature DCs are exposed to IL-10 [48], or when DCs are matured in the presence of IL-10 [19,20]. Importantly, IL-10-treated DCs acquire the ability to induce anergic T cells [48] with suppressive activity *in vitro*[19,20].

The tolerogenic effect of IL-10 on DCs is not simply due to the inhibition of proinflammatory cytokine production or of co-stimulatory molecule expression, but also to the induction or the expression/overexpression of tolerogenic molecules. IL-10 upregulates the production of IL-10 itself [49], the expression of HLA-G [50], of ILT2 and ILT4 [51], and of HO-1 [52,53] on DC precursors, rendering them regulatory cells capable of dampening immune responses and of inducing Tregs.

In addition to preventing DC activation, IL-10 modulates DC differentiation. The generation of CD1a^+^ human monocyte-derived DCs is impaired by the addition of IL-10 throughout the culture, and the resulting cells display a macrophage-like cell phenotype [54]. Other studies reported that treatment of human monocytes with IL-10 gives rise to a population of cells expressing markers associated with DCs such as CD83 and BDCA-3 [55,56]. We recently developed a protocol to efficiently differentiate a population of human tolerogenic DCs, DC-10, by culturing human monocytes in the presence of IL-10 (see next paragraph) [21].

### DC-10 are a distinct population of human tolerogenic dendritic cells

**Figure 1:**
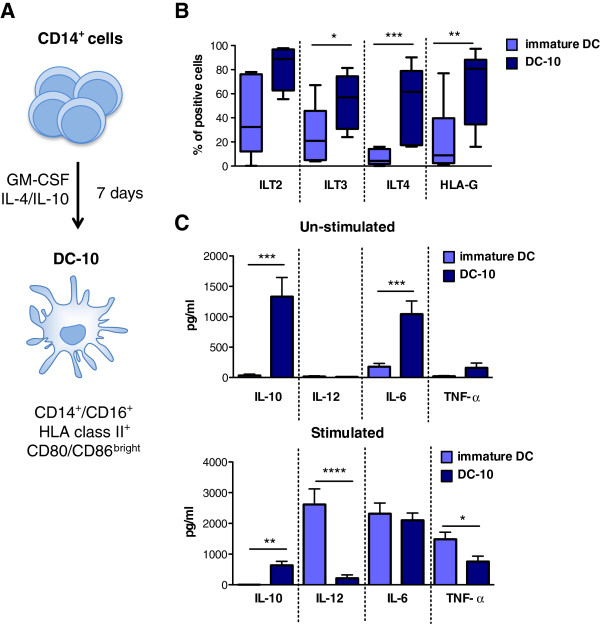
**Distinctive features of *in vitro *differentiated (tolerogenic) DC-10.** (**A**) Protocol for *in vitro* differentiation of DC-10. Peripheral blood monocytes are cultured for 7 days in the presence of granulocyte–macrophage colony-stimulating factor (GM-CSF), IL-4 and IL-10. (**B**) DC-10 express high levels of HLA-G and immunoglobulin-like transcript (ILT) 4. DC-10 were analyzed by flow cytometry to determine the levels of expression of ILT2, ILT3, ILT4 and HLA-G. Percentages of immature dendritic cells (DCs) and DC-10 expressing the tolerogenic molecules ILT2, ILT3, ILT4 and HLA-G (mean ± standard error, *n* = 16) are shown. (**C**) Cytokine secretion profile of immature DCs and DC-10 left unstimulated (upper panel) or stimulated with lipopolysaccharide and IFNγ (lower panel). Culture supernatants were collected after 48 hours and levels of cytokines were measured by ELISA (mean ± standard deviation, *n* = 16). As control, immature DCs differentiated by culturing monocytes with GM-CSF and IL-4 for 7 days were used. *P < 0.05; **P < 0.005; ***P < 0.0005; ****P < 0.0001.

Our group set up a protocol for the *in vitro* differen-tiation of human tolerogenic DCs, called DC-10, for their ability to spontaneously secrete large amounts of IL-10 [21]. DC-10 are differentiated from peripheral blood monocytes cultured for 7 days in the presence of GM-CSF, IL-4 plus IL-10 (Figure 1A). Resulting DC-10 are CD11c^+^CD11b^+^, express CD14 and CD16 but not CD1a, and, although not activated, display a mature myeloid phenotype, being CD83^+^, CD86^+^ and HLA-DR^+^. Moreover, DC-10 express high levels of HLA-G and of other signaling tolerogenic molecules ILT2, ILT3, and ILT4 (Figure 1B). In addition to spontaneously secrete high amounts of IL-10 (mean ± standard error 1.3 ± 0.3 ng/ml, *n* = 16; Figure 1C), DC-10 produce IL-6 (1.04 ± 0.2 ng/ml, *n* = 16), low levels of TNFα (0.16 ± 0.08 ng/ml, *n* = 16), and no IL-12. Importantly, DC-10 are phenotypically and functionally stable since, upon activation, they maintain their cytokine secretion profile (high IL-10/IL-12 ratio; Figure 1C) and their phenotype (S. Gregori and D. Tomasoni, personal communication). Functional assays showed that although DC-10 have a low capability to stimulate naïve CD4^+^ T cells, they induce the differentiation of anergic allo-specific IL-10-producing Tr1 cells [21], even upon activation (S. Gregori and D. Tomasoni, personal communication).

Comparative analysis demonstrated that DC-10 are phenotypically different and more potent than immature DCs [57] or IL-10-modulated mature DC (IL-10 DCs) [20] to promote allo-specific Tr1 cells. Both immature DCs and IL-10 DCs are indeed CD1a^+^CD14^–^, and express significantly lower levels of HLA-DR, CD80, CD83, and CD86 than DC-10 [58]. Immature DCs spontaneously secrete low levels of IL-10, while both IL-10 DCs and DC-10 secrete high amounts of IL-10 [58]. Upon activation, both immature DCs and IL-10 DCs produce significant amounts of IL-12 and TNFα, while DC-10 do not [58] (Figure 1C). Finally, stimulation of allogeneic naïve CD4^+^ T cells with immature DCs, IL-10 DCs, or DC-10 promotes the induction of Tr1 cells; however, at least three repetitive stimulations of naïve T cells with immature DCs or IL-10 DCs are required to induce suppressor Tr1 cells *in vitro* ([57] and S. Gregori, personal communication), whereas only a single stimulation of allogeneic naïve T cells with DC-10 is sufficient to generate a population of T cells comprising up to 15% of allo-specific Tr1 cells [59,60].

The high expression levels of ILT4, HLA-G, and IL-10 are necessary for the tolerogenic activity of DC-10 and their ability to prime T cells to become Tr1 cells. Indeed, addition of blocking antibodies against IL-10R, ILT4, or HLA-G during co-culture of DC-10 and naïve T cells completely prevented Tr1 cell induction [21]. This observation has been indirectly confirmed by studies in which we compared the ability of G-CSF and IL-10 to promote the induction of human tolerogenic DCs. G-CSF is a modulator of T-cell and DC functions. Previous reports showed that monocytes from G-CSF-treated healthy donors differentiate into tolerogenic DCs in the presence of autologous serum, which contains high levels of IL-10 and IFNα, and induce Tr1 cells *in vitro*[61]. This study demonstrated that G-CSF indirectly modulates DC functions. We recently defined a direct effect of G-CSF on DCs. Addition of G-CSF and IL-4 during monocyte-derived DC differentiation gives rise to a population of cells (G-DCs) that express CD14 and CD16, but not CD1a, display a mature myeloid phenotype, being HLA-DR^+^CD80^+^CD83^+^CD86^+^, and express the tolerogenic markers ILT4 and HLA-G [16], resembling the DC-10 phenotype. However, compared with DC-10, G-DCs produce lower levels of IL-10 and IL-6 if not stimulated, and higher levels of IL-12 and TNFα upon stimulation; moreover, G-DCs express significantly lower levels of HLA-G and ILT4 compared with DC-10 (M. Rossetti and S. Gregori, personal communication). Consistent with these findings, G-DCs retain hypostimulatory capacity but are not able to induce anergic and suppressive Tr1 cells [16].

DC-10 also differentiate Ag-specific Tr1 cells in autologous settings. We demonstrated that DC-10 from monocytes of allergic patients pulsed with allergen efficiently promote the generation of allergen-specific Tr1 cells able to suppress cytokine production by effector T-helper type 2 cells *in vitro*[58].

DC-10 are thus a population of tolerogenic DCs that can be easily differentiated and can be used to promote Ag-specific Tr1 cells *in vitro.*

### Comparison between DC-10 and other regulatory antigen-presenting cells under clinical development

**Figure 2:**
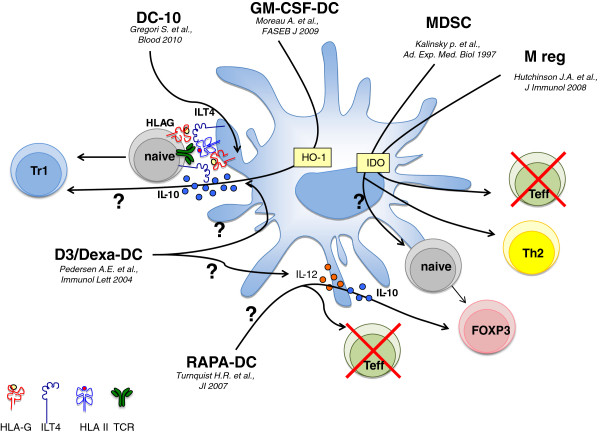
**Mechanisms of tolerance mediated by different tolerogenic antigen-presenting cells currently under clinical development.** DC-10 secrete IL-10 and upregulate the expression of immunoglobulin-like transcript (ILT) 4 and HLA-G molecules. During T-cell priming, ILT4/HLA-G interaction promotes anergy in T cells, which become type 1 regulatory T (Tr1) cells. Granulocyte–macrophage colony-stimulating factor (GM-CSF) dendritic cells (DCs) expressed heme oxygenase-1 (HO-1), which is involved in their mechanisms of action. It still remains to define whether HO-1 expression promotes IL-10 production by GM-CSF DC and Tr1 cell differentiation. Myeloid-derived suppressor cells (MDSCs) express indoleamine-2,3-dioxigenase (IDO) and induce T-helper type 2 skewing. Regulatory macrophages (M reg) are IDO^+^ and induce apoptosis of effector T cells while sparing forkhead box P3 (FOXP3)^+^ regulatory T cells (Tregs). Rapamycin (RAPA) DCs promote FOXP3^+^ Tregs, but the mechanism of induction is still under debate. 1,25-Dihydroxyvitamin D3/dexamethasone DCs promote suppressor T cells, but it is not clear whether this is mediated by ILT4 expression and IL-10 secretion.

Comparison between DC-10 and other regulatory APCs described in the literature, and currently under clinical development, indicates that DC-10 represent a population of human tolerogenic DCs with a unique phenotype and function (Figure 2). Despite the expression of CD14 and CD16, monocyte-derived DC-10 differ from human type 2 macrophages (M2 cells) generated *in vitro* from monocytes after exposure to M-CSF and IL-4 or IL-13, or IL-10 [62-64] (Table 1). Both DC-10 and M2 cells secrete high levels of IL-10 and low amounts of IL-12, but DC-10 produce IL-6, whereas M2 cells do not [62,64].

**Table 1:** Major characteristics of tolerogenic antigen-presenting cells

**Regulatory APCs**	**Precursor**	**Differentiation protocol**	**Adherent cells**	**Markers**	**Co-stimulatory molecules**	**Cytokines**	**Tolerogenic molecules**	**Mode of action**	**References**
DC-10	CD14^+^	GM-CSF,	No	CD1a^neg^	High	IL-10^high^	HLA-G^high^	IL-10	[21]
		IL-4,					ILT4^high^		
		IL-10 for 7d		CD14		IL-12^neg/low^		HLA-G	
				CD16 CD83		IL-6		ILT4	
						TNFα^low^			
M2 cells	CD14^+^	M-CSF,	Yes	CD68	Low	IL-10	Arginase	Soluble factors	[62,63]
		IL-4 or IL-13							
		for 6d		CD163					
				MR					
	CD14^+^	M-CSF,	Yes	CD68	Low	IL-10 TGF-β	Arginase	Soluble factors	[62,63]
		IL-10							
		for 6d		CD14 MR					
GM-CSF DC (macaque)	CD14^+^	GM-CSF for 7d	Yes	CD68	High	IL-10	HO-1	HO-1	[65,66]
	BM			CD14^neg^		IL-12^neg/low^			
RAPA-DC	CD14^+^	GM-CSF,	No	CD68	Low	IL-12^+^		IL-12	[67,68]
		IL-4,		CD14^neg^					
		RAPA for 7d				IL-10^low^			
						IL-6^low^			
D3/Dexa DC	CD14^+^	GM-CSF,	No	CD1a	Low	IL-10	ILT4	Possibly via IL-12	[69,70]
		IL-4,		CD14^neg^		IL-12^low^			
		for 9d							
		+Dexa (at d7)							
		+LPS and D3 (at d8)							
M reg	CD14^+^	M-CSF	Yes	CD64^+^	HLA-DR^+^	IL-6^low^	IDO	IDO	[71,72]
				CD14^neg/low^					
		for 6d		CD16^neg^	CD80^–/low^	IL-10^neg^			
				CD163^neg/low^		TNF-α^neg^			
									
		+ IFNγ (o.n.)			CD86^+^				
MDSC	CD14^+^	GM-CSF,	No	CD1a^neg/low^	Low	IL-10	IDO	PGE_2_	[26,28]
		IL-4,				TGF-β			
		PGE_2_ for 6d		CD14^+^			Arginase,		
							COX_2_		

APC, antigen-presenting cell; BM, Bone Marrow; COX2, cyclooxygenase-2; Dexa, dexamethasone; GM-CSF, granulocyte–macrophage colony-stimulating factor; HO-1, heme oxygenase-1; IDO, indoleamine-2,3-dioxigenase; ILT, immunoglobulin-like transcript; LPS, lipopolysaccharide; M2 cell, human type 2 macrophage; M reg, regulatory macrophage; M-CSF, macrophage colony-stimulating factor; MDSC, myeloid-derived suppressor cell; MR, mannose receptor; o.n., overnight; PGE_2_, prostaglandin E_2_; RAPA, rapamycin; TGF-β, transforming growth factor beta; D3, 1,25-dihydroxyvitamin D_3_.

DC-10 are different from tolerogenic DCs generated from nonhuman primate bone-marrow precursors with GM-CSF (GM-CSF DCs) [65] (Table 1). GM-CSF DCs have been extensively studied in nonhuman primates and rodents [65,66]; more recently a protocol to generate these cells from human peripheral blood monocytes has been developed (A. Moreau, The ONE Study Workshop, 2012). GM-CSF DCs are phenotypically different from DC-10 since they are CD68^+^ adherent cells and do not express DC-SIGN. Nonhuman primate and rodent GM-CSF DCs display a cytokine production profile that mirrors the one of DC-10, and express HO-1, a critical molecule used by these cells to actively modulate immune responses [66,73] (Figure 2). It still remains to be determined whether GM-CSF DCs generated from human peripheral blood monocytes are superimposable to those obtained from nonhuman primate and rodent bone-marrow precursors.

A protocol to differentiate human tolerogenic DCs using RAPA has been recently developed by the group of Angus W. Thomson ([67] and C. Macedo, The ONE Study Workshop, 2012) (Table 1). Addition of RAPA during monocyte-derived DC differentiation gives rise to a population of DCs termed RAPA-DC that are CD1a^+^CD14^–^, display an immature phenotype [32], and express low levels of the tolerogenic molecules ILT2, ILT3, and ILT4 [68]; RAPA-DC are thereby phenotypically different from DC-10. Interestingly, it has been shown that addition of RAPA at the beginning of DC differentiation prevents the ability of resulting DCs to secrete IL-12 upon activation [74]. However, when RAPA is added briefly before DC maturation, it inhibits IL-10 production with the concomitant increase of IL-12 [74]. Similar to DC-10, RAPA-DC weakly stimulate T cells and induce T-cell hyporesponsiveness [32,33]. In contrast to DC-10, however, RAPA-DC promote apopotosis of effector T cells and expand FOXP3^+^ Tregs [74] (Figure 2).

Treatment of immature DCs with 1,25-dihydroxyvitamin D_3_ in combination with IFNα upregulates the expression of both ILT3 and ILT4, and downregulates co-stimulatory molecules on resulting DCs, which, similarly to DC-10, acquired the ability to generate suppressor T cells via ILTs [75] (Table 1). Activation of immature DCs with 1,25-dihydroxyvitamin D_3_ and dexamethasone also promotes the induction of a population of DC (D3/Dexa-DC) that express ILT4 and low levels of co-stimulatory molecules, and secrete high levels of IL-10 in the absence of IL-12 [69,70]. D3/Dexa-DC inhibit T-cell proliferation and promote the induction of suppressor T cells (Figure 2). Interestingly, it has been demonstrated that cytokine polarization of naïve T cells into IL-10-producing T cells and anergy induction by D3/Dexa-DC were reverted by the addition of exogenous IL-12, whereas neutralization of IL-10 had no effects [70]. The lack of IL-12, and not the high levels of IL-10, is thus a key feature of D3/Dexa-DC regulatory activity.

The group of Edward K. Geissler developed a clinical grade protocol to generate human regulatory macrophages (M reg) from monocytes cultured with M-CSF and activated with IFNγ ([71] and J.A. Hutchinson and P. Riquelme, The ONE Study Workshop, 2012) (Table 1). M reg are CD14^–/low^CD16^–^CD64^+^CD163^–/low^HLA-DR^+^CD80^–/low^CD86^+^[71]. M-reg express IDO, which is involved in their regulatory functions [72], and studies performed with murine Mreg demonstrated that, when co-cultured with T cells, they induce apoptosis of effector T cells, while sparing FOXP3^+^ Tregs [76]. M-reg are thus phenotypically and functionally different from DC-10 (Figure 2).

Differentiation of monocytes in the presence of GM-CSF, IL-4, and prostaglandin E_2_ allows the induction of a population of regulatory APCs, termed myeloid-derived suppressor cells (MDSCs) ([26,28] and O. Natasa, The ONE Study Workshop, 2012) (Table 1). Myeloid-derived suppressor cells are CD1a^–^ cells, display a mature phenotype, and secrete IL-10 but not IL-12 [26], and thus they are phenotypically similar to DC-10. However, activation of naïve T cells in the presence of MDSCs promotes the induction of T-helper type 2 cells [77].

Overall, DC-10 share some similarities with other tolerogenic APCs but represent a unique subset of tolerogenic DCs characterized by the co-expression of high levels of ILT4 and HLA-G, with the distinct property of inducing Tr1 cells via the IL-10-dependent ILT4/HLA-G pathway.

### Clinical applications of tolerogenic DC-10

The identification of DC-10 as APCs that efficiently promote the induction of Tr1 cells *in vitro* prompted us to develop an efficient and reproducible *in vitro* method to generate, with minimal cell manipulation, allo-specific Tr1 cells, using DC-10 [59,60]. Activation of allogeneic T cells with DC-10 induces a population of alloAg-specific T cells (IL-10-anergized T cells) containing Tr1 cells that are anergic and actively suppress alloAg-specific effector T cells present within the mixed population. The protocol has been validated in good manufacturing practice (GMP) conditions. A pilot clinical trial for adoptive transfer of *ex-vivo* IL-10-anergized Tr1 cells of donor origin using DC-10 (or monocytes + IL-10; IL-10 DLI) has been completed in patients affected by hematological malignancies, who underwent T-cell-depleted haploidentical hematopoietic stem cell transplantation (ALT-TEN protocol) [78,79]. In this clinical setting, DC-10 *in vitro* differentiated from peripheral monocytes of patients have been used to anergize donor T cells. The goal of the trial was to provide immune reconstitution without severe graft-versus-host disease in the absence of immunosuppression. This first proof-of-concept clinical trial demonstrated the safety and feasibility of this approach. No acute adverse effects related to IL-10-anergized donor T-cell infusion were observed; however, rapid and long-term immune reconstitution together with absence of relapse were achieved [79]. The IL-10 DLI cell therapy can be used for the treatment of cancer patients and those with genetic hematologic diseases in the need of allogeneic hematopoietic stem cell transplantation, not only from haploidentical donors but also from matched unrelated donors. Moreover, the cell therapy protocol with IL-10-anergized Tr1 cells can be extended to prevent rejection after organ transplants. In this case, DC-10 *in vitro* differentiated from donor monocytes will be used to anergize recipient T cells. This cell product has been selected to be a part of The ONE Study, an integrated European Union-funded project, led by Edward K. Geissler in Regensburg (Germany). This cooperative project aims at developing and testing different subsets of regulatory cell products in kidney-transplanted recipients, allowing a direct comparison of the safety, clinical practicality and therapeutic efficacy of each cell type [80,81].

Ag-specific IL-10-anergized Tr1 cells can be induced with autologous DC-10 pulsed with a given antigen [58], thereby DC-10-derived Tr1 cells can be used as a cell product for restoring tolerance in autoimmune diseases.

Alternative to the use of DC-10 as inducers of Ag-specific Tr1 cells for tailoring Treg-based cell therapy, DC-10 represent an interesting therapeutic tool for DC-based cell therapy promoting and restoring tolerance in T-cell-mediated diseases. The challenge for the adoptive DC therapy is to generate tolerogenic DCs with a stable phenotype, which are resistant to maturation mediated by proinflammatory mediators. Recently, a comparative analysis of GMP protocols to generate human tolerogenic DCs using IL-10, TGFβ, 1,25-dihydroxyvitamin D_3_, dexamethasone or RAPA showed that IL-10 DCs are the most stable cell product. Based on these results the authors suggested that IL-10 DCs are the best suitable subset of tolerogenic DCs for tolerance-inducing therapies [82]. We showed that DC-10 are phenotypically stable cells and are functionally more efficient than IL-10 DCs in promoting Ag-specific Tr1 cells *in vitro* (S. Gregori and D. Tomasoni, personal communication), thereby representing a good candidate for cell-therapy approaches. In this scenario, the use of allogeneic DC-10 or autologous DC-10 pulsed with a given Ag can be predicted to prevent graft rejection or restore tolerance in T-cell-mediated diseases, such as autoimmune diseases and allergy.

## Conclusions and perspectives

Since their discovery, DCs have proved to play a central role in regulating immune responses. Moreover, significant advances have been made in establishing methods to manipulate DCs *in vitro* to generate tolerogenic DCs suitable for clinical applications. In this scenario, DC-10 are a good candidate since they can be easily differentiated *in vitro* from monocyte precursors, and are stable cells with potent suppressive functions. A protocol to generate Ag-specific Tr1 cells by DC-10 for adoptive Treg-based cell therapy has been developed and validated in GMP for clinical purposes. Moreover, DC-10 are of great potential interest as a therapeutic tool *per se* to induce or re-establish immunological tolerance in different clinical settings including allogeneic transplantation or autoimmune diseases. The manufacturing protocol for GMP production of DC-10 is under development. Further studies in humanized mouse models and in large animal models are warranted to establish the best route and dose of administration, lifespan and homing kinetics of DC-10, in order to design clinical protocols to test the safety and efficacy of DC-10-based cell therapy.

## Abbreviations

Ag: antigen; APC: antigen-presenting cell; DC: dendritic cell; IDO: indoleamine-2,3-dioxigenase; FOXP3: forkhead box P3; G-CSF: granulocyte colony-stimulating factor; GM-CSF: granulocyte–macrophage colony-stimulating factor; GMP: good manufacturing practice; HO-1: heme oxygenase-1; IFN: interferon; IL: interleukin; ILT: immunoglobulin-like transcript; M-CSF: macrophage colony-stimulating factor; RAPA: rapamycin; TGF: transforming growth factor; TNF: tumor necrosis factor; Tr1: type 1 T regulatory; Treg: regulatory T cell.

## Competing interests

Both authors declare that they have no competing interests.

## Authors’ contributions

GA performed some experiments and contributed to the preparation of the manuscript. SG conceived the scientific idea, supervised the project and prepared the manuscript. All authors read and approved the final manuscript.
